# Isolated Avulsion of the Common Hepatic Duct from Blunt Abdominal Trauma

**DOI:** 10.1155/2012/254563

**Published:** 2012-07-05

**Authors:** Victor W. Wong, Arvin Gee, Paul Hansen, Andrew Michaels

**Affiliations:** ^1^Department of Surgery, Oregon Health and Science University, 3181 SW Sam Jackson Park Road, Portland, OR 97239, USA; ^2^Department of Surgery, Legacy Emanuel Medical Center, 2801 N. Gantenbein, Portland, OR 97227, USA; ^3^Portland Providence Cancer Center, 4805 NE Glisan Street, Portland, OR 97213, USA; ^4^The Oregon Clinic, 4805 NE Glisan Street, Portland, OR 97213, USA

## Abstract

Isolated extrahepatic biliary tract injury following blunt abdominal trauma is rare. The underlying pathogenic mechanisms remain obscure, but include shear and/or compression forces on the biliary system. Associated morbidity rates are high and largely the result of delays in diagnosis. Imaging modalities commonly employed for diagnosis include ultrasonography, computed tomography, nuclear medicine, and magnetic resonance imaging. Percutaneous and endoscopic techniques have been used both for diagnosis and treatment. Treatment options are dictated by the stability of the patient and the extent of bile duct and concomitant injuries. In this paper, we discuss a case of isolated avulsion of the hepatic duct confluence following blunt trauma that was successfully managed with Roux-en-Y hepaticojejunostomy. To our knowledge, this specific injury pattern has not been previously reported.

## 1. Introduction

Isolated biliary tract injury following blunt abdominal injury is extremely rare [1]. The mechanism of injury remains unclear but has been attributed to shearing and/or compression forces on the extrahepatic biliary system. Morbidity rates of up to 40% have been reported, largely the result of delays in diagnosis [2–6]. Here we report a case of isolated avulsion of the hepatic duct confluence following blunt trauma that was successfully managed with Roux-en-Y hepaticojejunostomy.

## 2. Case Report

**Figure 1:**
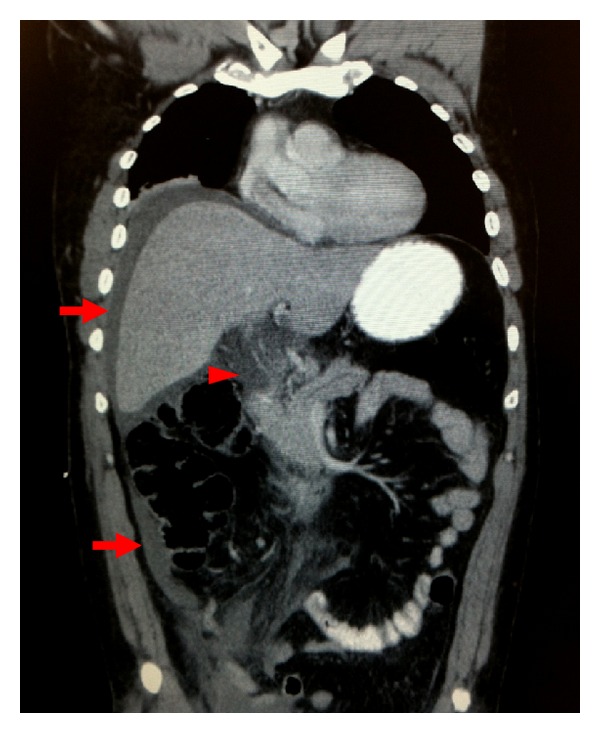
Coronal CT image demonstrating intraabdominal fluid above the liver and along the right pericolic gutter (arrows). “Periportal tracking” is also present (arrowhead), suggestive of an extrahepatic biliary injury. Possible disruption of the extrahepatic bile duct is visualized.

A 45-year-old man sustained blunt compression injury to his abdomen while working under a van. He was immediately extricated and presented at a referring hospital 24 hours later with mild abdominal fullness and pain. Upon transfer to our hospital, he had developed worsening abdominal pain and one episode of nonbilious emesis. He was tachycardic (120 beats/min) with mildly elevated blood pressure (140/80). His abdomen was distended, diffusely tender; and without signs of external trauma. Abnormal laboratory studies included WBC 19×10^3^/*μ*L, ALT 86 units/L, alkaline phosphatase 136 units/L, and total bilirubin 3.7 mg/dL. Contrast-enhanced computed tomography (CT) of the chest/abdomen/pelvis revealed free fluid around the liver and right pericolic gutter (Figure 1) without evidence of extraluminal air or solid organ injury. Clips from a laparoscopic cholecystectomy (performed three years earlier for acute cholecystitis) were visualized in the hepatic fossa. Given the high suspicion for bowel and/or bile duct injury, he was taken to the operating room for exploratory laparotomy.

**Figure 2:**
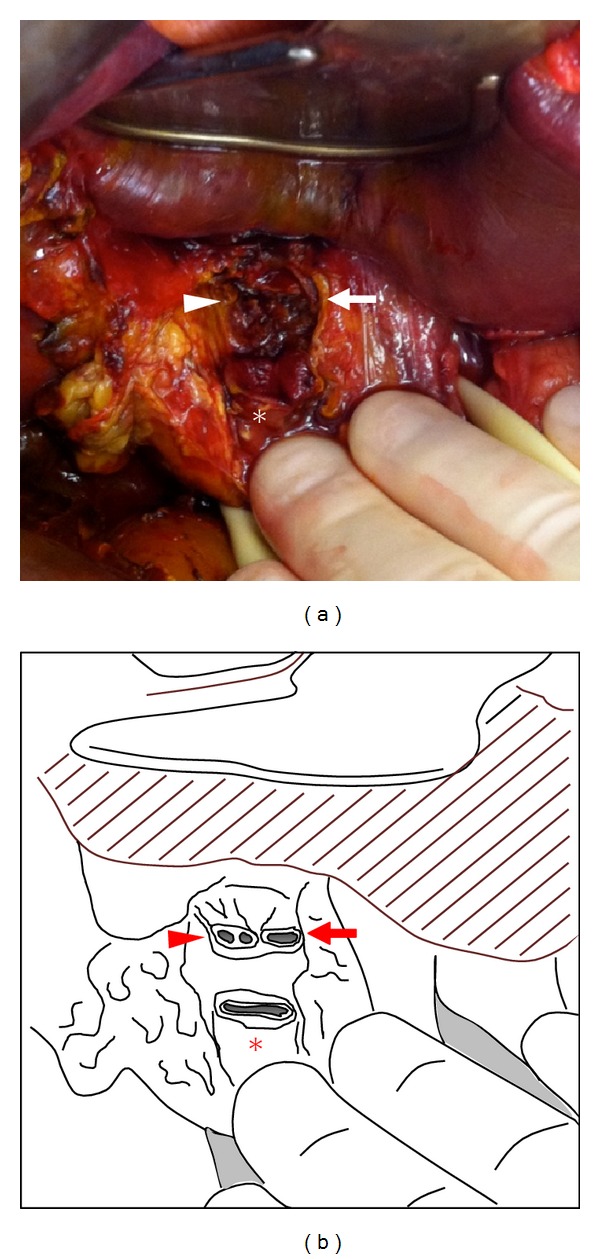
Intraoperative photograph (a) and schematic (b) demonstrating isolated avulsion of the hepatic duct confluence. The right anterior and posterior hepatic ducts (arrowhead), left hepatic duct (arrow), and common hepatic duct (asterisk) are visualized.

Exploration revealed bilious fluid that tracked into the right pericolic gutter and retroperitoneum. No injuries were detected in any solid organs or hollow viscera. Medial rotation of the right colon and duodenum did not reveal injuries in the lesser sac or retroperitoneum. The hepatic hilum was bile stained and dissection of the hepatoduodenal ligament demonstrated complete avulsion of the hepatic duct at the confluence. The left hepatic duct and the right posterior and anterior duct junctions were visualized (Figure 2(a)). The common hepatic duct stump was oversewn and a single-layer hepaticojejunostomy was created using a retrocolic Roux-en-Y limb (Figure 2(b)). A stapled side-to-side jejunojejunostomy was performed and two closed suction drains were placed at the biliary-enteric anastomosis. The patient was discharged home with a single closed suction drain that was removed six weeks postoperatively. He has continued to do well at over three months after injury.

## 3. Discussion

Although isolated bile duct injuries have been described, isolated avulsion of the common hepatic duct confluence following blunt trauma has not been previously reported. Extrahepatic biliary tract injury after blunt trauma is exceedingly rare and often associated with injuries in adjacent organs [4, 6–9]. The diagnosis is often delayed and may be missed when the initial exploration reveals other injuries. A high index of suspicion is the key to prompt identification and treatment of extrahepatic bile duct injuries.

Several mechanisms have been proposed for blunt injury of the extrahepatic biliary system. These can be grouped into three categories: (1) crushing against the rigid spinal column, (2) shearing against areas of relative fixation, and (3) rapid emptying of a distended gallbladder into the bile ducts.

Our patient had a prior laparoscopic cholecystectomy, suggesting that rapid compression of a filled gallbladder is not an essential factor in injuries of this type. Another hypothesis proposes that shearing forces lift the liver superiorly while the hepatoduodenal ligament is pulled inferiorly [10]. The lack of concomitant arterial or venous injury in our patient remains puzzling but may be attributed to the relative elasticity and redundant length of the portal vein and proper hepatic artery compared to the bile ducts [1].

Diagnostic investigations commonly reported include ultrasonography and CT imaging [11]. The presence of “periportal tracking” on CT may indicate injury to the extrahepatic biliary system but is nonspecific [12]. Percutaneous aspiration may reveal bilious fluid and isotope (hepatobiliary iminodiacetic acid-HIDA) scintigraphy has been used to diagnose extrahepatic bile leaks but lacks the ability to precisely identify the injury site [11, 13]. Magnetic resonance cholangiopancreatography (MRCP) can provide high resolution imaging of the hepatic hilum and has been used to identify biliary injury following blunt liver trauma [14]. Percutaneous transhepatic cholangiography (PTC) can also be utilized to precisely define the injury but is invasive and may be difficult to perform in nondilated bile ducts [13]. Endoscopic retrograde cholangiopancreatography (ERCP) is a valuable diagnostic option in the stable patient and is increasingly employed for the treatment of extrahepatic biliary injury [15].

Surgical options for extrahepatic ductal injuries depend on the stability of the patient [16]. In hemodynamically unstable patients, the operative goals should be external drainage and possibly primary repair for injuries involving <50% of the duct circumference [17]. More significant injuries in an unstable patient can be treated with a bridging T-tube across the injured segments and definitive repair at a later time. Stable patients with complete transaction of an extrahepatic bile duct should undergo biliary-enteric anastomosis with a Roux-en-Y limb [18]. Primary end-to-end duct repair is associated with a higher long term rate of stricture [3]. Injury to the main hepatic ducts can also be treated with a Roux-en-Y hepatoportoenterostomy, hepatic resection, or ligation of the right or left hepatic duct. Early surgical complications include infection and anastomotic leak. In the long term, stricture formation can result in recurrent cholangitis, biliary cirrhosis, and portal hypertension. Morbidity and mortality (up to 40% in some reports) are highly associated with the presence of concomitant injuries and delays in diagnosis.

To our knowledge, isolated avulsion of the hepatic duct confluence following blunt abdominal compression injury has not been previously reported. A high index of suspicion is critical to effectively identify and manage these injuries. Upon diagnosis, treatment is dictated by the hemodynamic stability of the patient and the extent of injury. Although various endoscopic, percutaneous, and surgical options are available, biliary-enteric reconstruction provides the best long term outcome for major injuries to the extrahepatic biliary system.
